# Unveiling the hidden players: noncoding RNAs orchestrating polyamine metabolism in disease

**DOI:** 10.1186/s13578-024-01235-3

**Published:** 2024-06-25

**Authors:** Marianna Nicoletta Rossi, Cristian Fiorucci, Paolo Mariottini, Manuela Cervelli

**Affiliations:** Department of Sciences, University of Roma Tre, 00146 Rome, Italy

**Keywords:** Polyamines, Noncoding RNA, Gene expression, Polyamine metabolism

## Abstract

Polyamines (PA) are polycations with pleiotropic functions in cellular physiology and pathology. In particular, PA have been involved in the regulation of cell homeostasis and proliferation participating in the control of fundamental processes like DNA transcription, RNA translation, protein hypusination, autophagy and modulation of ion channels. Indeed, their dysregulation has been associated to inflammation, oxidative stress, neurodegeneration and cancer progression. Accordingly, PA intracellular levels, derived from the balance between uptake, biosynthesis, and catabolism, need to be tightly regulated. Among the mechanisms that fine-tune PA metabolic enzymes, emerging findings highlight the importance of noncoding RNAs (ncRNAs). Among the ncRNAs, microRNA, long noncoding RNA and circRNA are the most studied as regulators of gene expression and mRNA metabolism and their alteration have been frequently reported in pathological conditions, such as cancer progression and brain diseases. In this review, we will discuss the role of ncRNAs in the regulation of PA genes, with a particular emphasis on the changes of this modulation observed in health disorders.

## Polyamines generalities

Polyamines (PA) are aliphatic compounds with amino groups at both ends of their molecular structure, found widely in almost all living organisms. These small polycations play a vital role in driving proper cell proliferation and differentiation in prokaryotes [1–3] and eukaryotes [4]. Among PA the most prevalent in animal cells are putrescine (Put), spermidine (Spd), and spermine (Spm). Under physiological pH condition, PA are positively charged and form weak associations with negatively charged intracellular molecules, including nucleic acids, phospholipids, and ATP. Of particular interest, PA exhibit a higher affinity for RNA, thereby influencing protein translation and inducing alterations in mRNA structure [5].

Due to their essential role in cell growth, PA are notably abundant in actively proliferating cells [6], in fact, in tumour cells PA metabolism is often dysregulated, indicating that their elevated content is necessary for transformation and tumour progression [7, 8]. Recently, new data are elucidating the mechanisms through which PA can establish a tumour-permissive microenvironment [9]. Notably, PA appear to exert a pivotal influence on the regulation of antitumour immune response, which becomes unresponsive to the immune checkpoint blockade, leading to the existence of immunologically ‘cold’ tumours [9]. Interestingly, PA levels can be subjected to alteration based on factors such as microbiota composition, dietary PA availability and tissue’s responsiveness to its local microenvironment, contributing to tumour progression [9]. Natural PA can prevent oxidative damage to DNA and phospholipids by functioning as free radical scavengers [10–16]. In fact, increase level of catabolic PA enzymes sensitizes tumour cells to irradiation [17–20]. In brain, PA play a pivotal role during the development and as modulators of different ion channels [21–24]. Indeed, intracellular Spm acts at µM concentrations as a significant blocker of inwardly rectifying potassium channels (Kir) and a rise of its content causes an increase in channel gating and rectification, which in turn leads to cellular excitability of neurons and muscle fibers [25]. Recently an additional mechanism of regulation on glutamate ionotropic channels has been described. This mechanism involves ancillary proteins such as TARP, cornichons, neuropilin and tolloid-like proteins (NETOs) that attenuate channel blockage allowing PA to exit the pore. It follows that the permeation of PA occurs at membrane potentials that are more negative and therefore more physiologically relevant [24]. Polyamines also have the capacity to regulate the assembly of certain acetylcholine receptors containing negatively charged amino acids within the α4 or α7 cytosolic loop [26] and control the functioning of glutamate receptors, including NMDA, AMPA and kainate [21, 27]. Despite their recognized role in brain, it is important to note that PA are unable to pass the brain–blood barrier (BBB) [28, 29]. Polyamine transport into the brain involves a variety of transport mechanisms, such as large pores like connexins and pannexin hemichannels, as well as specific transporters like polyspecific organic cation transporters (OCTs) belonging to the solute carrier (SLC) 22A1-3 family. These transporters play a crucial role in facilitating the entry of PA [29, 30]. Polyamines are important also in the regulation of diabetes. Indeed, Spd and Spm can also interact with insulin-like growth factor-1, promoting an increase in pro-insulin gene transcription and regulating insulin signalling [31]. Moreover, PA exert their function in diabetes in part by regulating hypusination process. Hypusination is a post-translational modification of a conserved lysine residue of the translation factor eIF5A that depends on the presence of Spd [32]. Indeed, hypusination is essential for proper development of the exocrine pancreas as well as endocrine function, indicating that a scarcity of hypusinated eIF5A (eIF5Ahyp) has detrimental consequences [33]. However, an overabundance of eIF5Ahyp exacerbates the hallmarks of the diabetic phenotype. In general, elevated levels of PA are reported in both exocrine and endocrine cells of the pancreas, which may contribute to endoplasmic reticulum stress, oxidative stress, inflammatory response, and autophagy [34]. Finally, supplementation with either Spd or Spm has been shown to effectively enhance glucose homeostasis and insulin sensitivity [35]. Given their pleiotropic roles, intracellular PA levels need to be kept within a specific range, that is crucial for optimal cellular function, by balancing their transport in and out of cells and metabolism. The physiological levels of PA can vary depending on the tissue and/or cell type. For instance, in mouse brain tissues, physiological level of Put is estimated to be around 10 nmol/g, while both Spm and Spd contents are near 250/300 nmol/g [36]. Conversely, in C2C12 murine myoblasts, PA are less concentrated, with Put level ranging around 1 nmol/g, while both Spm and Spd contents are in the range of 10 nmol/g [37].

### Polyamine metabolism and transport

Given the multifaceted functions of PA, the regulation of PA homeostasis through biosynthesis, catabolism and transport is very strict. The enzymes and transporters responsible for controlling intracellular PA pools are subject to stringent control mechanisms operating at various levels, including transcription, translation, and degradation. Each level of regulation possesses its own feedback mechanisms that specifically responds to alterations in intracellular PA levels. Consequently, the dysregulation of PA metabolic enzymes can have adverse effects on human health, including the development of conditions such as cancer, muscle disease, and neurodegeneration [30, 38–40].

**Fig. 1:**
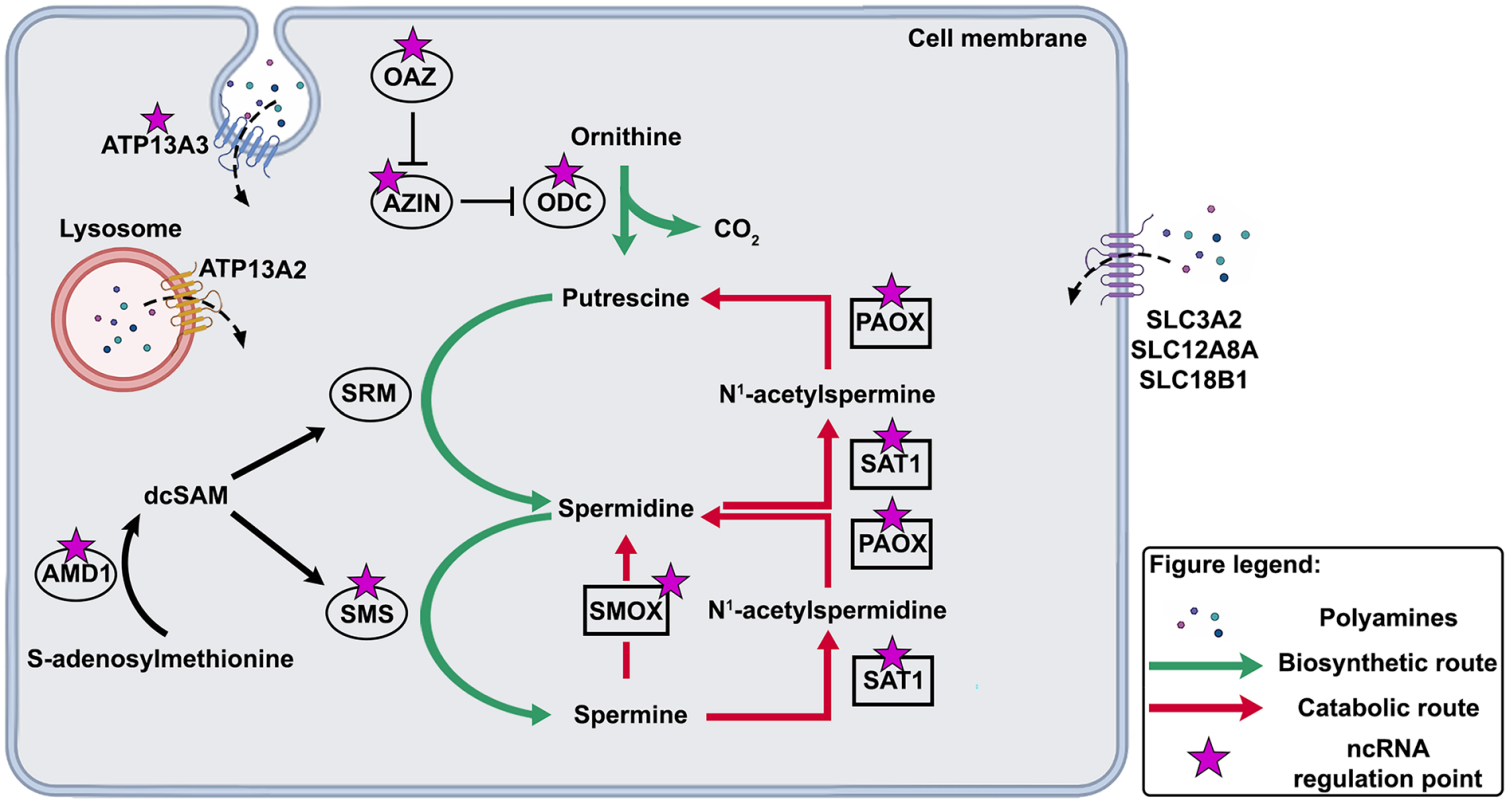
Schematic diagram of polyamine metabolism. Biosynthetic and catabolic pathways are shown in green and in red respectively. Stars highlight the presence of a ncRNA regulation

Polyamines biosynthesis is regulated by the enzymes ornithine decarboxylase (ODC), which catalyses the conversion of Ornithine into Put, and by two distinct aminopropyl transferases, Spd synthase (SRM) and Spm synthase (SMS), which add an aminopropyl group to Put and Spd, respectively (Fig. 1). ODC is the first rate-limiting enzyme in PA biosynthesis and various mechanisms, including transcription, mRNA stability, translation, and degradation, tightly regulate ODC levels to quickly adapt to cellular requirements [41]. Three antizymes (OAZ1, OAZ2 and OAZ3) and two antizyme inhibitors (AZIN1 and AZIN2) mediates the post-translational control of ODC [42]. OAZ inhibits ODC dimerization and promotes its proteasomal degradation [43]. On the other hand, antizyme inhibitors, homologous proteins to ODC but lacking enzymatic activity, interact with OAZs even more efficiently than ODC itself, thereby counteracting their inhibitory effect [44, 45]. Another key enzyme in PA biosynthesis is S-adenosylmethionine decarboxylase (AMD1), responsible for generating decarboxylated S-adenosylmethionine (dcSAM), the aminopropyl donor for SRM and SMS. On the other hand, spermidine/spermine N^1^-acetyltransferase (SAT1), peroxisomal N^1^-acetyl-spermine/spermidine oxidase (PAOX), and spermine oxidase (SMOX) are the three enzymes of PA catabolism [18, 46, 47]. Both Spm and Spd receive acetyl groups from acetyl-coenzymeA at the N^1^ position via the enzyme SAT1, resulting in the formation of N^1^-acetylspermidine and N^1^-acetylspermine, respectively. Subsequently PAOX oxidises these substrates to produce the final products Spd and Put, as well as 3-aceto-aminopropanal (3-AAP) and hydrogen peroxide (H_2_O_2_) [48, 49]. The third catabolic enzyme SMOX directly oxidises Spm to produce Spd, 3-aminopropanal (3-AP) and H_2_O_2_ [48]_._

Polyamines are imported into cells through multiple PA transport systems (PTSs) [50]. There are two proposed mechanisms for PA import: transport through the plasma membrane and endocytic routes. Transporters located on the plasma membrane belong to the solute carrier (SLC) and ATP-binding cassette (ABC) families [51–53]. Among the SLC transporters, only SLC18B1 has undergone biochemical validation as a PA transporter. SLC18B1 is a vesicular transporter (VPAT) with widespread expression in humans, particularly in the lung, placenta, and adrenal gland. Biochemical studies using purified SLC18B1 protein in proteoliposomes have demonstrated its active transport of Spd and Spm [54]. SLC3A2, a plasma membrane transporter also called DAX, has been found involved in exporting Put and importing arginine in human colorectal carcinoma cells [55].The *SLC12A8A* gene encoding the cation-chloride cotransporter 9 isoform a (CCC9a), is widely expressed in mammals and is primarily localized in intracellular compartments. However, certain splice variants can reach the plasma membrane. Overexpression of *SLC12A8A* gene in HEK cells has been shown to enhance the uptake of PA (mainly Spd) and aminoacids [56]. Additionally, in the brain, PA are synthesized endogenously in neurons and then exported to astrocytes [29].

Recent studies have identified a novel family of transporters that play a crucial role in the mammalian PA uptake pathway, potentially operating in conjunction with the previously proposed endosomal PA uptake pathways. This newly identified family includes two widely distributed transporters known as ATPase cation transporting 13A2/3 (ATP13A2 and ATP13A3), which belong to the P5B-ATPase family [53, 57, 58]. It has been demonstrated that ATP13A2 transport PA, exhibiting the highest affinity for Spm and Spd [53].

Interestingly, OAZs and AZINs are able to modulate the PA plasma membrane transport in a negative and positive fashion, respectively [59, 60].

Alteration of PA metabolism can result from physiological stimuli as well as from pathological conditions like cancer, inflammation, and neurodegeneration [38, 40, 61–63]. Aberrant PA metabolism has been reported in diabetic patients and animal models of diabetes. In a clinical study in patients, serum Put has been found significantly elevated in patients with type 2 diabetes (T2D) compared to those without diabetes and Spm was significantly associated with fasting insulin levels. Moreover, serum Put and Spm levels were associated with a higher risk of T2D [64]. Polyamines have been shown to play a role in the development of diabetic complications, such as diabetic nephropathy, by promoting inflammation and fibrosis in the kidneys [34]. A recent study [65] demonstrated that PA biosynthesis inhibition can safeguards β-cell function. This conclusion stems from a comprehensive approach that involved integrated experiments with human β-cell-specific knockout mice, in vitro cultures of human islets, and a multicentre clinical trial. The findings suggest that such inhibition could offer a safe strategy to enhance survival rates among individuals with recent-onset type 1 diabetes [65].

Abnormal PA metabolism has also been linked to the development and progression of various types of cancer. Among the PA-related genes, *ODC* is in several cases overexpressed [66, 67], while *SAT1* could be both down- or up-regulated [9, 68–70]. Moreover, while AZs have been reported to function as tumour suppressor and to negatively regulate tumour cell proliferation and transformation [71], the AZIN1 RNA editing, a post transcriptional modification that enhances its activity, has been found higher in many different cancers spanning from hepatocellular carcinomas to lung cancer and is associated to increased transformation and tumorigenesis [72, 73]. Furthermore, abnormal PA metabolism has been implicated in the pathogenesis of several neurodegenerative diseases, including Snyder–Robinson syndrome, Bachmann–Bupp syndrome, and Parkinson’s disease (PD). The Snyder–Robinson syndrome is a genetic condition that is linked to a mutation in a PA metabolic gene. This X-linked mental retardation and developmental disorder is brought on by mutations in the SMS gene that is located on chromosome X in the Xp22.1 region [74]. Bachmann–Bupp Syndrome is inherited in an autosomal dominant way and is associated to ODC1 pathogenic variant. Bachmann–Bupp Syndrome is characterized by behavioural abnormalities, feeding difficulties, hypotonia, alopecia, global developmental delay in the moderate to severe range [75]. A broad range of studies, encompassing human patients, yeast, and mouse models, have provided substantial evidence supporting the role of defects in the PA pathway in the development of PD [76, 77]. Prior research has suggested a link between PA and PA metabolic enzymes, particularly a decrease in SAT1, and the increased aggregation of α-synuclein [77], a hallmark of PD. Furthermore, mutations in ATP13A2 gene, also known as PARK9 have been associated with Kufor–Rakeb Syndrome, an early-onset variant of PD [78]. There is the proposal that dysfunctional lysosomal PA export may serve as a mechanism underlying lysosome-dependent cell death, potentially contributing to neurodegeneration [78]. Moreover, AZIN2 has been found increased in brains affected by Alzheimer’s disease [79, 80] and its depletion leads to a reduction in Put levels, which is associated with alterations in motor function. These observations imply a role for AZIN2 in the regulation of dopaminergic neuron function [81].

Among the mechanisms that fine-tune regulate PA metabolic enzymes, emerging findings highlight the importance of ncRNAs and the present review will deal with microRNA, long noncoding RNA e circRNA in the regulation of PA metabolism.

## Noncoding RNA: microRNA, lncRNA and circRNA

The discovery of the first noncoding RNA (ncRNA) with regulatory function dates back to 1988 when a small bacterial RNA from *Escherichia coli* was identified, capable of regulating the transcription of the *micF* gene [82]. Since then, genomic studies have revealed a large amount of DNA that is transcribed but not translated, leading to the description of hundreds of regulatory ncRNAs [83–85].

The importance of ncRNA is emphasized by the observation that an increase in the number of ncRNAs correlates with the evolution of vertebrate complexity [86–88].

**Fig. 2:**
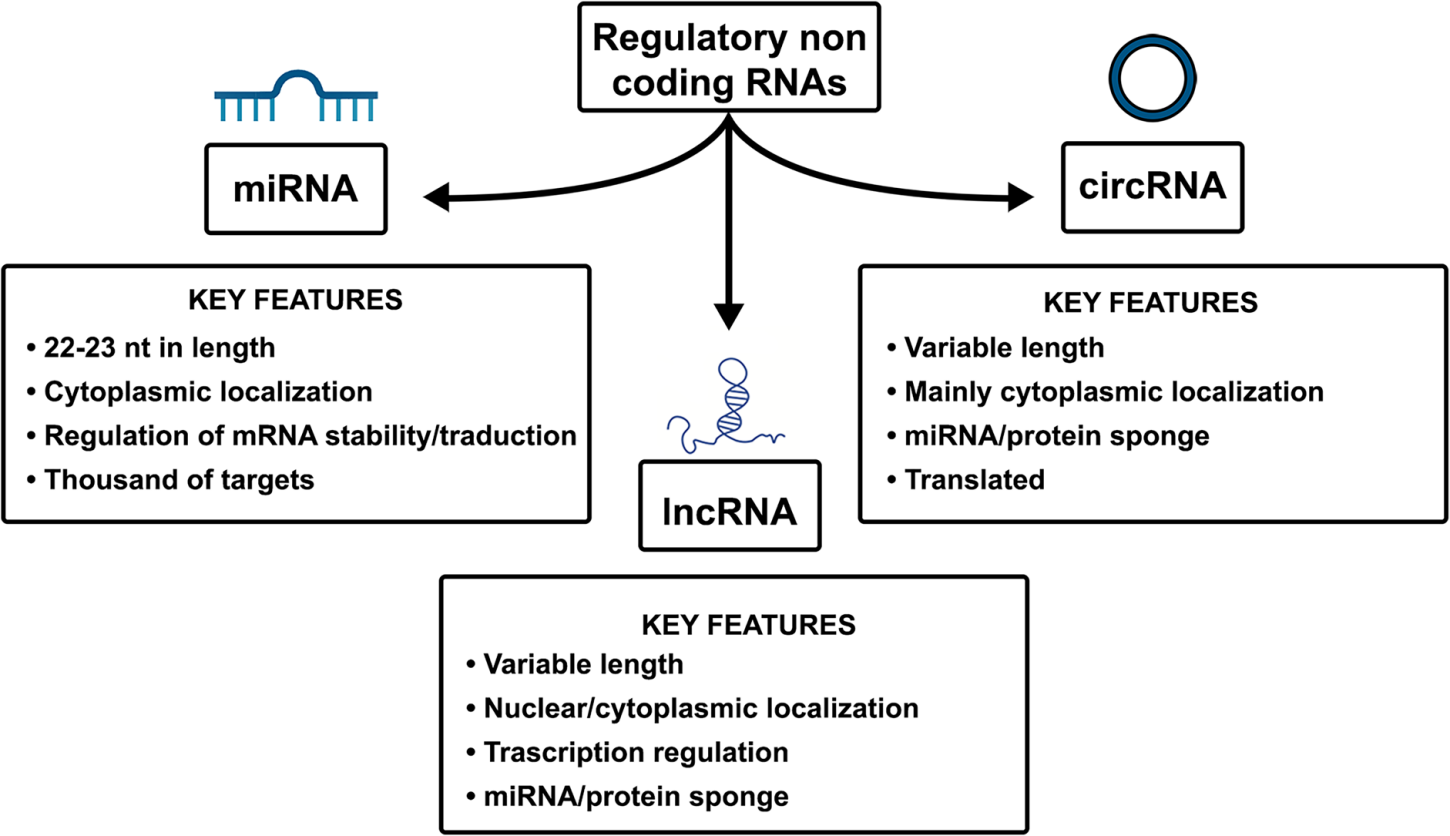
Schematic diagram of regulatory noncoding RNA classification. The key characteristics of each group are reported in the squared boxes

Regulatory ncRNAs can be divided into three main classes based on their length and structure: (1) short ncRNA, which are less than 200 nt in length; this class includes snoRNA, snRNA, piRNA and microRNA (miRNA); (2) long ncRNAs, which exceed 200 nt in length; (3) circular RNAs (circRNAs), characterized by their circular structure, with variable nucleotide lengths (Fig. 2). Noncoding RNAs take part in the regulation of many biological processes, from proliferation to differentiation and cell death [89, 90]. Indeed, they are emerging as key regulators of chromatin accessibility, transcription, post-transcriptional regulation, and protein synthesis. Through their activities, ncRNAs can drive the expression/repression of many cellular targets with high tissue, cell and time specificity [91–94].

MicroRNAs are single-stranded ncRNAs of 22 nucleotides able to post-transcriptional regulate gene expression by binding to target mRNAs. Their corresponding genes are transcribed as miRNA precursors called pri-miRNA by RNA Polymerase II (RNAPol II) and their transcripts are spliced, polyadenylated and further maturated to give duplex miRNA of 22 nt [95]. One of the two filaments (the guide strand) is translocated to the RNA-induced silencing complex (RISC) where it binds to a target mRNA, leading to the degradation of the complementary strand. The RISC complex can guide the mRNA silencing through different mechanisms, depending also on the grade of complementarity between the mRNA and the miRNA. In particular, the mRNA-miRNA pairing results in one of the following events: (1) cutting of the mRNA strand, leading to degradation; (2) destabilization of the mRNA through the shortening of its polyA tail; (3) repression of translation. Imperfect pairing of miRNAs with the 3′ untranslated region (3′UTR) of the target mRNA cause a blockade of mRNA translation, while perfect matching leads to mRNA degradation [96]. MicroRNAs can catalyse multiple rounds of RNA cleavage therefore amplifying their effects. High complementarity with the 5′ end of the miRNA seed region seems to be crucial for the recognition of target mRNAs, whereas pairing at the 3′ end of the miRNA can be more variable [97, 98]. Moreover, a single miRNA can have different targets in a biological pathway and on the other hands, a single gene can be targeted by multiple miRNA [96].

Since the identification of the first miRNA, let4, in *Caenorhabditis elegans* [99, 100], which plays a crucial role in worm development, our understanding of the roles of miRNAs has continued to increase. Nowadays, there are plenty of documentation indicating that miRNA participate in the regulation of many cellular processes and diseases, ranging from development to cancer (for a review see [101]).

Long noncoding RNAs (lncRNAs) are a very heterogeneous group of molecules that vary in size, subcellular location, and function. Their lengths range from few hundred nucleotides (*e.g.* 340 nucleotides of 7SK) to several thousands (*e.g.* 90 kb of kcnq1ot1). They can be transcribed by RNA polymerase II or III, and be either spliced or not [102]. Regarding the subcellular localization, lncRNAs can be localized in the nucleus or in the cytoplasm. Nuclear lncRNAs are usually regulators of transcription and of chromatin architectures, able to bind transcription factors, chromatin remodelers and specific regulatory regions on promoters and enhancers [93, 103, 104]. For example, some nuclear lncRNAs recruit gene silencing complexes, like PRC1 and PRC2 to target gene promoters. Other lncRNAs, such as GAS5, act as decoys, precluding the access of regulatory proteins to DNA [105]. In contrast, cytoplasmic lncRNAs are involved in post-transcriptional control, working as miRNA sponge, regulating the abundance and activity of specific miRNAs, or modulating mRNA stability, by forming duplexes with the 3′UTRs of target mRNAs [106, 107]. Due to their different mechanisms of action, lncRNAs are involved in many pathophysiological processes, spanning from cellular differentiation and pluripotency to the development of cancer.

Circular RNAs are covalently closed single strand molecules of RNA that lack the characteristic signature of many cellular lncRNAs and mRNAs such free ends, 5′cap and polyadenylated tail. They arise from back-splicing of exon-exon junctions of pre-mRNAs. CircRNAs can vary in length, ranging from less than 200 nt to more than 3000 nt [108], and may contain one or more exons, as well as introns. The generation of circRNAs is dependent on the presence of both cis- and trans-acting factors. Among the cis-acting factors, complementary sequences (such as Alu repeats) in the flanking introns are necessary for the back-splicing mechanism. Trans acting factors can influence circRNA levels through various mechanisms: (1) affecting the probability of base pairing of reverse complementary sequences [*e.g.* ADAR1, which mediates adenine to inosine (A-to-I) conversions]; (2) stabilizing the double-strand RNA duplex (*e.g.* NF90/NF110); (3) or disrupting the double-strand RNA duplex (*e.g.* DHX9) [109].

Circular RNAs are usually more stable of their linear counterpart [110]. Their formation is dynamically regulated in a tissue-specific and developmentally dependent manner [92]. The precise mechanisms governing circRNA degradation are not yet fully understood and appear to be multifaceted. Degradation mechanisms may involve the action of Ago2, RNase L (a cytoplasmic endonuclease) or, in the case of circRNA modified with the N6-methyladenosine (m^6^A), the ribonuclease complex RNase P/MRP, mediated by the proteins like methyladenosine RNA binding protein F2 (YTHDF2) and reactive intermediate imine deaminase A homolog (RIDA) [111].

The most documented role of circRNA is their function as sponge for miRNAs, although they have also been reported to act as sponges for RNA binding proteins. A specific class of intronic circRNAs retained in the nucleus can act as transcriptional regulators by interacting with U1 small nuclear ribonucleoprotein (snRNP), RNAPol II, and the promoters of their parental genes to enhance transcription. The importance of circRNA is especially crucial in brain development and function, as they are notably enriched in brain compared to other organs and a major fraction of circRNA derive from genes expressed in neurons [112].

Accumulating evidence demonstrated that circRNAs could encode functional polypeptides and are actively translated through mechanisms independent of 5′capping, such as those mediated by internal ribosomal entry sites (IRESs) or m^6^A modification [113, 114]. In conclusion, miRNAs, lncRNAs, and circRNAs are involved in fine-tuned mechanisms controlling gene transcription and protein expression and frequently their function involves intricate interactions among them forming an endogenous RNA network. These complex networks comprise not only lncRNA/miRNA, circRNA/miRNA interactions but also lncRNA/miRNA/circRNA interactions, amplifying the complexity of the regulation. This crosstalk between different ncRNAs is particularly significant in the brain, where it regulates not only neuronal differentiation but also neuronal plasticity and synaptogenesis [109]. Moreover, perturbation of levels and interactions of the ncRNAs could lead to pathologic events like apoptosis, inflammation, and neurodegeneration. In the following paragraphs the involvement of miRNAs, lncRNAs and circRNAs in the regulation of PA genes is specifically discussed.

## Noncoding RNA regulation of polyamine metabolism in cancer

As reported above, PA play a central role in cell proliferation, and it is not surprising that changes in their levels and the activity of their metabolic enzymes have been frequently described in many cancer types [115]. In details, SMS dysregulation was associated with carcinogenesis and in particular its high expression was related to poor survival and increased risk of metastasis in triple-negative breast cancer [116]. In breast cancer, Chen and co-workers [117] identified a specific regulatory mechanism in which the loss of miR-3613-3p leads to an increase in SMS mRNA levels (Table 1). The miR-3613-3p gene is often deleted in tumour samples, and bioinformatic analysis identified SMS as one of its target mRNAs. This regulatory effect of miR-3613-3p on SMS has been validated in breast cancer tissues [117].

**Table 1:** Regulation of genes involved in polyamine metabolism by ncRNA. Genes are reported in alphabetic order

Gene	ncRNA	Cell/tissue type	Disease	Model organism	Effect	References
Adenosylmethionine decarboxylase 1 (AMD1)	miR-762	Embrionic stem cell	–	Mouse	↓	[134]
Antizyme inhibitor 1 (AZIN1)	miR-433	Cardiac fibroblasts	Cardiac fibrosis	Mouse	↓	[149]
	circNFIB	Cardiac fibroblasts	Cardiac fibrosis	Mouse	↑	[149]
	circMap4k2	Heart left ventricule, cardiomyocytes, fibroblasts	Heart failure	Mouse	↑	[159]
	miR-106a-3p	Heart left ventricule, cardiomyocytes, fibroblasts	Heart failure	Mouse	↓	[159]
	MALAT1	Liver immortalized cell lines	Ischemia–reperfusion (IR)	Mouse	↑	[152]
	miR-150-5p	Liver immortalized cell lines	Ischemia–reperfusion (IR)	Mouse	↓	[152]
ATPase cation transporting 13A2 (ATP13A3)	miR-130/301	Primary endothelial cells	Pulmonary arterial hypertension	Human	↓	[142]
N^1^-acetyl-spermine/spermidine oxidase (PAOX)	KIKAT/LINC01061	Kaposi’s sarcoma associated herpesvirus cell lines	Kaposi’s sarcoma	Human	↑	[127]
Ornithine decarboxylase antizyme 2 (OAZ2)	miR-34a	Colorectal cancer tissue and cell lines	Colorectal cancer	Human	↓	[119]
Ornitine decarboxylase (ODC1)	miR378a	Colorectal cancer tissue and cell lines	Colorectal cancer	Human/mouse	↓	[118]
Spermidine/spermine N^1^-acetyltransferase (SAT1)	miR-199a-5p	Pituitary adenomas tissue	Pituitary adenomas	Human/mouse	↓	[121]
	mir-139-5p, mir195, mir320c and mir34c-5p	Prefrontal cortex human	Psychiatric disease	Human	↓	[138]
	LINC00265	Osteosarcoma tissue and cell lines	Osteosarcoma	Human	↑	[124]
	ASMTL‐AS1	Lung adenocarcinoma cell lines	Lung adenocarcinoma	Human	↑	[125]
	lnc-HZ03/miR-hz03	Villous tissues/trophoblastic cells	Recurrent miscarriage	Human	↑	[153]
Spermine oxidase (SMOX)	miR-124	Gastric cancer cell lines	Gastric cancer	Human	↓	[120]
	mir-139-5p, mir195, mir320c and mir34c-5p	Prefrontal cortex human	Psychiatric disease	Human	↓	[138]
	circHIPK2	Neural stem cell	Brain ischaemic stroke	Mouse	↑	[139]
	LVBU	Colorectal cancer cell lines and tissue	Colorectal cancer	Human	↑	[126]
Spermine synthase (SMS)	miR-3613-3p	Breast cancer cell line and tissue	Breast cancer	Human	↓	[117]

In colorectal cancer, a more complex network has been identified, where miR-378a was found to inhibit ODC1 (Table 1) both directly by binding to its 3′UTR and indirectly by inhibiting a transcription factor that activates ODC1 transcription. In particular, miR-378a targets FOXQ1, which is responsible for activating the transcription of c-MYC, a key transcriptional activator of ODC1 [118].

The miR-378a is the most downregulated microRNA in colorectal cancer tissues, suggesting its potential protective and anti-proliferative role. Moreover, miR-378a induces apoptosis and inhibits proliferation and migration. By preventing the specific binding of miR-378a to ODC1 the authors demonstrated that ODC1 is essential to mediate miR378a anticancer activity. The activity of ODC1 can be modulated also indirectly by acting on OAZ and AZIN. Indeed, for example in colon cancer, it has been demonstrated that the OAZ2 mRNA stability is negatively regulated by miR-34a that directly targets OAZ2 3′UTR [119]. This can be one of the mechanisms through which miR-34a exerts some of its pro-tumoral functions.

In a gastric cancer cell model, miR-124 expression is significantly downregulated through epigenetic mechanisms, and a search for its targets identified SMOX mRNA (Table 1). Indeed, miR-124 directly binds to the 3′UTR of SMOX. Accordingly, SMOX activity is upregulated in adenocarcinoma cellular model and the use of a demethylating agent can restore miR-124 expression and downregulate SMOX [120].

In the pituitary adenomas, the second most common intracranial tumour, PA biosynthesis and in particular SAT1 expression has been found altered. A search for dysregulated microRNA in these tumours, compared to normal pituitary tissue, identified five microRNAs with abnormal expression. Among them, miR-199a-5p was significantly downregulated. To functional prove miR-199a-5p effect, the authors demonstrated that overexpression of miR-199a-5p suppressed cell differentiation and invasive behaviour of pituitary tumour cells. Interestingly, the miR-199a-5p overexpression downregulated SAT1 protein and mRNA levels (Table 1) [121].

In all the above-mentioned experimental settings microRNAs were found aberrantly downregulated in different tumours, but on the contrary the miR-210 has been found upregulated in clear cell renal carcinoma, [122], as well as in other malignancies [123]. To get inside in the functions of miR-210, the authors performed a metabolomic analysis in proximal tubular cells after miR-210 overexpression and interestingly, among the altered metabolites, they identified also the PA Put and Spd. Even if the authors did not address the specific target genes of miR-210, its impact on PA metabolism was clearly demonstrated [123].

Collectively in five different cancer models of both human and mouse origin, alterations in microRNA have been associated to key enzymes in PA metabolism, highlighting the important role of PA in sustaining cellular proliferation and cancer development.

Furthermore, also the role of lncRNAs in the regulation of PA metabolism has been investigated mainly in cancer models. In several reports, lncRNAs exert their effects interacting with and modulating the abundance of miRNA. For instance, in an osteosarcoma model, it has been described with bioinformatic and clinical approaches an interesting pathway where the lncRNA LINC00265 is overexpressed leading to a decrease of the miR-382-5p. Among the validated miRNA targets there is also the enzyme SAT1 (Table 1) [124]. SAT1 expression has also been found modulated in lung adenocarcinoma by another lncRNA, named ASMTL‐AS1 (Table 1). In this context, ASMTL‐AS1 recruits the splicing factor U2AF2 to stabilize SAT1 mRNA, promoting ferroptosis [125]. It has to be noted that ASMTL‐AS1 is implicated in various tumour types, suggesting a potentially similar mechanism in other malignancies.

In colorectal carcinoma, a hypoxia-induced lncRNA LVBU is highly expressed and correlates with poor cancer prognosis. The effect of this lncRNA is exerted through the interaction with miR-10a/miR-34c, which protects the transcription factor B-cell lymphoma 6 (Bcl-6) from degradation. Bcl-6 in turn, inhibits p53-mediated suppression of genes involved in urea cycle and in PA synthesis, including ODC1 (Table 1). It is worth noting that urea cycle and PA metabolism alteration occur in various tumours, but the underlying deregulation mechanisms remain elusive. The induction of the lncRNA LVBU if confirmed in other cancers, could represent a common transforming mechanism and, therefore, a promising anti-cancer target [126]. It is interesting to note that p53 regulates PA metabolism by inducing the catabolic enzyme SAT1 and repressing the biosynthetic enzyme ODC.

Finally, a complex regulatory mechanism has been described in Kaposi’s sarcoma associated herpesvirus (KSHV). In this type of cancer, the lncRNA KIKAT/LINC01061 has been identified as a binding partner of KDM4A, a histone lysine trimethyl demethylase known as an oncogene in various cancer types. The methyl groups removal from H3K9me3 on a promoter region by KDM4A is associated with gene upregulation. The authors found that KIKAT/LINC01061 interaction with KDM4A may mediate relocalization of KDM4A at the transcription start site (TSS) of the promoter region, leading to the transactivation of target genes. Among the genes upregulated by KIKAT/LINC01061 there is the PA catabolic enzyme PAOX (Table 1). Indeed, on the promoter region of PAOX the overexpression of KIKAT/LINC01061 leads to a shift of KDM4A peak from –851 to –290 nt. The relocation of KDM4A mediated by KIKAT/LINC01061 on PAOX promoter could be an intriguing mechanism involved in the progression of Kaposi’s sarcoma [127].

## Noncoding RNA regulation of polyamine metabolism in brain

The role of PA and their metabolic enzyme in brain function, development and pathology has now emerged [29, 128–133]. The key biosynthetic enzyme Amd1 is regulated by miR-762 during neuronal progenitor cell differentiation, leading to a significant reduction in Amd1 protein levels (Table 1) [134]. The miR-762, interacting with the 3′UTR of Amd1, drives a shift in ribosomal load that leads to its translational repression. Mutational experiments confirmed that miR-762 is sufficient for Amd1 down-regulation [134]. Interestingly, an association between the noradrenalin/serotonin and glutamatergic neuronal circuits with PA has been investigated [128, 135, 136], revealing PA as possible protective molecules in brain, important to prevent the development of mental disorders and epilepsy [137]. Lopez and colleagues [138] have analysed the expression levels of SAT1 and SMOX and of some microRNAs that are predicted to target SMOX and SAT1 (miR-139-5p, miR-195, miR-320c and miR-34c-5p) in the prefrontal cortex of suicide completers compared to psychiatric healthy controls. Their findings demonstrated a significant correlation between these miRNAs and the expression levels of the PA genes (Table 1) [138], highlighting how microRNAs can have a key role in neurological diseases also through the regulation of PA genes expression.

A recent study explored the role of the circHIPK2 in neural stem cell (NSC) differentiation, a key process in brain development, neuronal plasticity, and post-ischaemic stroke recovery [139]. The study found that circHIPK2 expression is downregulated during NSCs differentiation, and silencing circHIPK2 appeared to downregulate SMOX expression (Table 1). This finding is intriguing because SMOX is an important mediator in the regulation of cerebral ischaemic injury [140], and circHIPK2 may participate in its transcriptional regulation in the context of brain ischaemic stroke.

## Noncoding RNA regulation of polyamine metabolism in other healthy and pathological conditions

Polyamines play a central role also in diabetes mellitus, as they prevent the upregulation of glucose and ketone and, similarly to insulin, counteract the disease [141]. Polyamines also enhance mitochondrial respiration and thereby regulate all major metabolic pathways. With the aim to explore the mechanisms underlying the deregulation of PA metabolism in diabetes, Kambis and colleagues [31] analysed all the overexpressed miRNAs in Diabetic Cardiomyopathy and observed their association with PA metabolism. Interestingly, in diabetes mellitus some deregulated microRNAs have been found associated to PA metabolism, among them miR-210 and miRNA-199a-5p.

Another study shows an interesting bidirectional relationship between miR-130/301 and the PA transporter ATP13A3 (Table 1) [142] in endothelial cells. In fact, forced miR-130a expression decreases ATP13A3, while the depletion of ATP13A3 induces an increase in miR-130/301 expression, suggesting a positive feedback loop that promotes endothelial cell apoptosis and pulmonary arterial hypertension [142].

Fibrosis is the final common pathological outcome of many chronic inflammatory diseases, could affect nearly every tissue in the body and eventually leads to organ malfunction and failure [143]. Several findings highlighted that AZIN1 have a role in regulating fibrosis in different organs, such as liver [144], heart [145] and kidney [146]. Mechanistically, decreased level of AZIN1 activated TGF-β1, the major profibrotic factor while AZIN1 overexpression suppressed TGF-β signalling and the fibrotic response [145]. The regulation of AZIN1, at least in the context of renal fibrosis is mediated by the miR-433, as the authors demonstrated that overexpression of miR-433 suppressed Azin1 expression [146].

Two recent papers enriched the picture featuring the involvement of circRNAs in the regulation of AZIN1 in cardiac fibrosis. Cardiac fibrosis plays a crucial role in the development and evolution of heart failure [147] and is a common pathological feature of most adverse cardiac events such as myocardial infarction and diabetic cardiomyopathy [148]. In this context, a first paper [149] identified a circRNA, named circNFIB able to positively regulate AZIN1 by sponging the miR-433, an important component of TGF-β/Smad3-signalling and a direct regulator of AZIN1. More recently, Yan and colleagues (2023) analysed the expression profile of circRNAs after surgical ventricular reconstruction (SVR), a therapeutic approach for heart failure, and identified circMap4k2 (named according to its mother gene, Map4k2) as the most upregulated circRNA. CircMap4k2 promotes cardiac regeneration by acting as microRNA sponge. They found that miR-106a-3p, known for regulation of cell growth and proliferation in tumours [150] binds to circMap4k2. Moreover, among the predicted targets of miR-106a-3p, AZIN1 was the only experimentally confirmed target. Thus, circMap4k2 by targeting the miR-106a-3p/AZIN1 pathway could enhance cardiomyocyte regeneration.

Ischemia–reperfusion (IR) is a common pathological process in various organs and in liver is an inevitable complication occurring during liver surgeries that involves a complex cascade of inflammatory mediators [151]. In a mouse model of liver IR, the LncRNA MALAT1 has been described to target the miR-150-5p. Looking for mRNA targets of miR-150-5p, the authors found AZIN1 [152] and demonstrated that AZIN1, miR-150-5p and MALAT1 constitute a competing endogenous RNA (ceRNA) network in this condition.

Finally, in villous tissues and in trophoblastic cells from women with recurrent miscarriage, the lnc-HZ03 and the miR-hz03 have been identified forming a positive feedback loop upregulating each other. The miR-hz03 could also enhance p53 levels by stabilizing its mRNA. The p53 protein, in turn, induces SAT1 and thus the authors propose that lnc-HZ03 and miR-hz03 are able to perturbate PA metabolism influencing cell viability and apoptosis [153].

## circRNAs generated from polyamine mRNA backsplicing

**Fig. 3:**
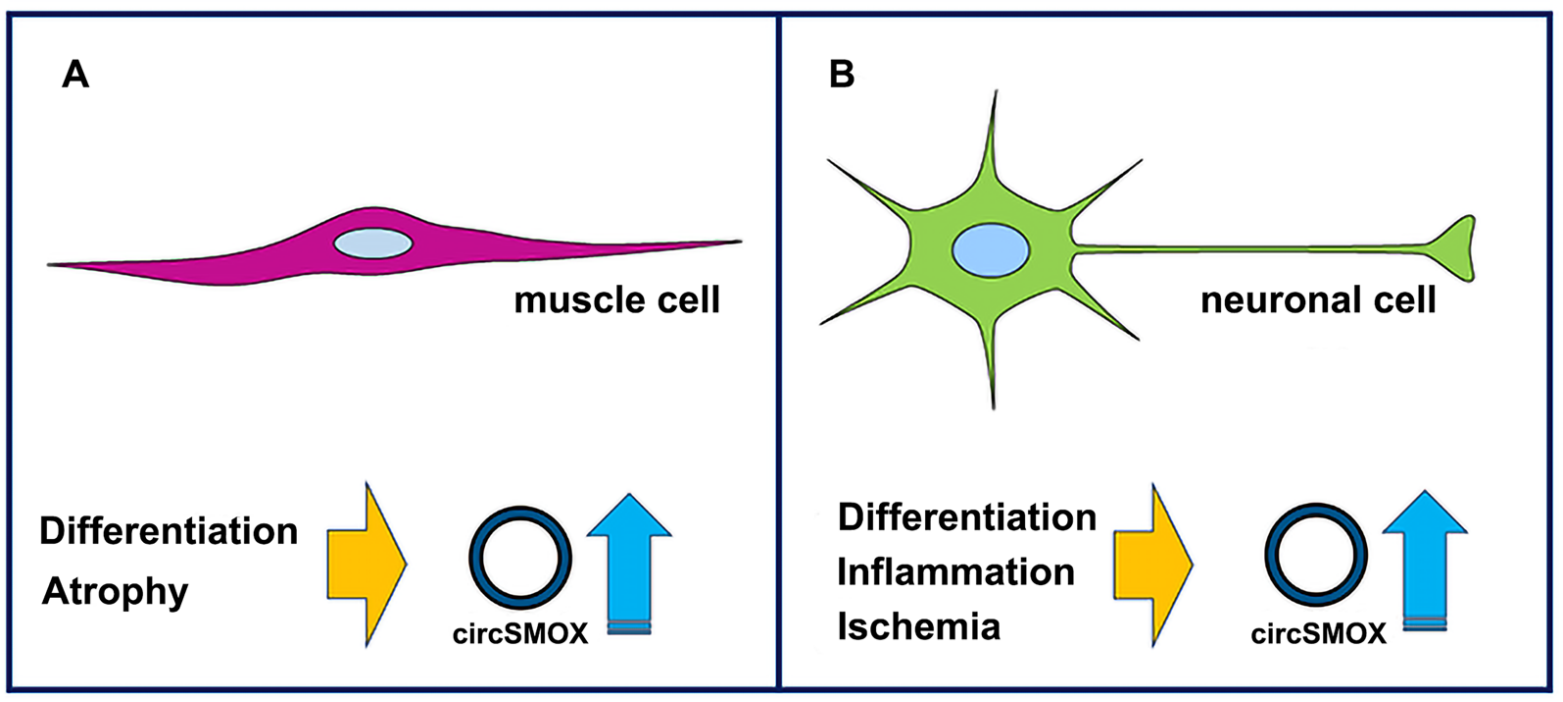
Schematic diagram of circSMOX expression in muscle (left panel) and neuronal (right panel) cells. The conditions were circSMOX has been found upregulated are reported. Blue arrows indicate upregulation of expression

It deserves a particular mention the circRNAs generated from backsplicing of PA metabolic gene transcripts. In recent years, unbiased RNAseq analysis allowed the scientific community to identify many circRNAs differentially expressed especially during brain development and in neurologic diseases [113]. The first annotation of a circular RNA from a PA gene was in mouse brain and ES cells samples by Memczak and co-workers [110] (Table 2). This analysis revealed a circRNA of 460 nt derived from *SMOX* gene, named circSMOX, which includes exon 2 and 3 of the linear SMOX transcript [110]. Afterwards, Ribak-Wolf and colleagues [92] analysed brain circRNAs during development to assess brain-specific circRNA expression, examining 29 datasets from both human and mouse. Interestingly, circSMOX was found in synaptosomes and in cytoplasm of mouse brain samples, during P19 cells differentiation (in particular at day 12) and during primary neuron maturation (where circSMOX was consistently detected from day 0 to day 28, with a pick at day 14). In 2017, an altered expression of circSMOX was described in mouse brain after transient focal ischemia by RNAseq analysis [154]. In 2018, the first indication of circSMOX expression in a tissue outside the brain was reported. It was found to be increased in a transcriptomic analysis of a mouse model of muscular atrophy [155]. The experimental validation and characterization of circSMOX was carried out in 2020, when its expression was analysed in atrophic C2C12 and in two mouse models of ALS [156]. Interestingly, during muscle differentiation, both linear and circular SMOX showed a similar pattern of distribution, reaching their peak at 96 h post induction of differentiation. However, under atrophic condition, a distinct and complementary expression profile emerged, with the linear SMOX transcript decrease and a parallel circSMOX increase. This differential expression of circSMOX was also demonstrated in two ALS mouse models, specifically FUS and G93A, during the progression of the disease [156]. This intriguing observation suggests a possible function of circSMOX in ALS etiopathology and, more broadly, in atrophic conditions (Fig. 3). To gain a deeper understanding of circSMOX, future analysis will be required to investigate its specific role, the mechanisms governing its expression, and the effects of circSMOX on its linear counterpart and PA metabolism. A recent study by Han and colleagues demonstrated that circSMOX plays a functional role in PC12 cell response to LPS stimulation [157]. Indeed, the authors demonstrated that the circRNA expression increases after stimulation and that circSMOX interacts with the miR‐340‐5p, functioning as a miRNA sponge. Notably, one of the targets of miR‐340‐5p is the protein SMURF1, an E3-ubiquitin ligase implicated in neuroinflammation [158].

**Table 2:** Evidence of circRNAs arising from genes involved in polyamine metabolism

Gene	circRNA	Length (nt)	Cell types	Organism	References
SMOX	circSMOX	460	Brain and ES cells samples	Mouse	[110]
			Brain (synaptosomes and cytoplasm) P19 cells (day 12 of differentiation) primary neuron	Mouse	[92]
			Transient focal ischemia	Mouse	[154]
			Model of muscular atrophy		[155]
			Atrophic C2C12 and in two mouse models of ALS		[156]
			PC12 cell lines	Rat	[157]
ODC1	hsa_circ_0052603	113	Occipital lobe	Human	[92]
	hsa_circ_0116927	124	SH-SY5Y differentiation		[92]
AZIN1	hsa_circ_0135367	115	SH-SY5Y differentiation	Human	[92]
	hsa_circ_0135368	1839	Frontal_cortex		
	hsa_circ_0135369	1979	Temporal_lobe		
	hsa_circ_0135370	193	Diencephalon, frontal_cortex, occipital_lobe, parietal_lobe		
	hsa_circ_0135371	1187	Frontal_cortex		
	hsa_circ_0135372	320	Cerebellum, occipital_lobe, parietal_lobe, temporal_lobe		
	hsa_circ_0135373	606	Frontal_cortex, occipital_lobe		
	hsa_circ_0135374	628	Occipital_lobe		
	hsa_circ_0135375	802	Diencephalon, frontal_cortex, temporal_lobe		
	hsa_circ_0135376	999	Cerebellum, diencephalon, frontal_cortex, temporal_lobe		
	hsa_circ_0085278	292	Cerebellum, diencephalon, parietal_lobe, temporal_lobe,		
	hsa_circ_0135377	465	Cerebellum, diencephalon, frontal_cortex, occipital_lobe		
	hsa_circ_0007374	639	Cerebellum, frontal_cortex, occipital_lobe, parietal_lobe, temporal_lobe		
	hsa_circ_0085280	308	Temporal_lobe		
	hsa_circ_0135378	690	Frontal_cortex, temporal_lobe		
	hsa_circ_0135379	172	Sy5y_exp1_D4, SY5Y_exp2_D4		
	hsa_circ_0135380	5846	Diencephalon		
	hsa_circ_0008921	455	Hs68_control, Hs68_RNase, diencephalon, K562		[92, 160, 161]
	hsa_circ_0004982	371	Hs68_control, Hs68_RNase, WAS2, frontal_cortex, parietal_lobe, Ag04450, Bj, Gm12878, K562, Mcf7		[92, 160–162] [92, 160–162]
	hsa_circ_0003304	544	Hs68_control, Hs68_RNase, platelets, cerebellum, diencephalon, frontal_cortex, occipital_lobe, parietal_lobe, temporal_lobe, Ag04450, Bj, Gm12878, H1hesc, Hepg2, Huvec, K562, Mcf7		
	hsa_circ_0085286	138	Diencephalon		[92, 161]
	mmu_circ_0005622	296	Frontal_cortex	Mouse	[92]
	mmu_circ_0005623	2001	Forebrain		
	mmu_circ_0005624	780	Forebrain, PN_D01, midbrain		
OAZ1	hsa_circ_0109303	173	Parietal_lobe	Human	[92]
OAZ2	hsa_circ_0104223	765	SY5Y_exp2_D8	Human	[92]
	hsa_circ_0104224	123	Diencephalon

Among the other genes codifying for PA metabolic enzymes, ODC1, OAZ1/2 and AZIN1/2 appear to give rise to circRNAs (Table 2). Specifically, two circRNAs, hsa_circ_0052603 of 113 nt and hsa_circ_0116927 of 124 nt, originating from the human ODC1 gene, have been annotated in the human brain [92]. Hsa_circ_0052603 was identified in the occipital lobe, while hsa_circ_0116927 was detected at day 2 of SH-SY5Y cell differentiation [92]. The existence of a circular RNA arising from the ODC gene is yet to be confirmed but given the significant role of ODC in cancer and in brain pathophysiology, its validation will pave the way for further studies aimed at understanding the regulation of ODC in cancer cells. Moreover, querying circRNA database (http://www.circbase.org/) for potential cirRNAs arising from OAZ1/2 and AZIN1/2 results in the identification of numerous potential back-splicing events (Table 2). Specifically, OAZ1 yielded one circRNA, hsa_circ_0109303, with a length of 173 nt, while OAZ2 produced two circRNAs, hsa_circ_0104223 and hsa_circ_0104224, measuring 765 and 123 nt, respectively (Table 2). Notably, the AZIN1 gene gave rise to twenty-one and three different circRNAs in human and mice samples, respectively. These circRNAs exhibited variable length spanning from 115 nt of hsa_circ_0135367 to 5846 nt of hsa_circ_0135380. Interestingly, one of them, the circRNA hsa_circ_0135374 is conserved also in mice with the name mmu_circ_0005624 [92]. The conservation of this circRNA between human and mouse suggests a potentially crucial conserved function, warranting further investigation. To sum up, among the circRNAs generated from backsplicing of PA metabolic gene transcripts, only circSMOX has been experimentally validated, but, considering the roles that circRNAs have in physiology and pathophysiology, the presence of circRNAs arising from polyamine genes needs to be deeply investigated. Future studies will help to confirm the presence of circRNAs from PA genes, to understand their roles and their influence on the expression of the linear counterpart.

## Conclusions

The intricate networks of ncRNAs involved in the regulation of PA metabolism are only beginning to be uncovered. Given the multifaceted roles of PA, it would not be surprising to discover that numerous others ncRNAs are involved in controlling PA biosynthetic and catabolic enzymes. Currently, the emerging picture reveals a multi-layered, tissue-specific regulations of PA metabolic enzymes, which is often disrupted or loss in pathological conditions, particularly in cancers (Table 1). Moreover, it is important to note that many ncRNAs have the characteristic of co-regulating different genes and pathways. This feature allows to link the PA metabolism to various cellular processes, integrating different stimuli to generate a comprehensive and reliable cellular response. Finally, this complex network of noncoding RNA interacting with PA metabolism also paves the way for new opportunities in therapeutic intervention in the fields of neurological and neuromuscular diseases, diabetes and cancer.

## Data Availability

Not applicable.

## References

[1] Tabor CW, Tabor H (1985). Polyamines in microorganisms. Microbiol Rev.

[2] Kurihara S, Suzuki H, Oshida M, Benno Y (2011). A novel putrescine importer required for type 1 pili-driven surface motility induced by extracellular putrescine in *Escherichia Coli* K-12. J Biol Chem.

[3] Michael AJ (2016). Polyamines in eukaryotes, bacteria, and archaea. J Biol Chem.

[4] Pegg AE (2009). Mammalian polyamine metabolism and function. IUBMB Life.

[5] Lightfoot HL, Hall J (2014). Endogenous polyamine function—the RNA perspective. Nucleic Acids Res.

[6] Mandal S, Mandal A, Johansson HE, Orjalo AV, Park MH (2013). Depletion of cellular polyamines, spermidine and spermine, causes a total arrest in translation and growth in mammalian cells. Proc Natl Acad Sci.

[7] Casero RA, Murray Stewart T, Pegg AE (2018). Polyamine metabolism and cancer: treatments, challenges and opportunities. Nat Rev Cancer.

[8] Chia T-y, Zolp A, Miska J (2022). Polyamine immunometabolism: central regulators of inflammation, cancer and autoimmunity. Cells.

[9] Holbert CE, Cullen MT, Casero RA, Stewart TM (2022). Polyamines in cancer: integrating organismal metabolism and antitumour immunity. Nat Rev Cancer.

[10] Khan AU, Di Mascio P, Medeiros MH, Wilson T (1992). Spermine and spermidine protection of plasmid DNA against single-strand breaks induced by singlet oxygen. Proc Natl Acad Sci U S A.

[11] Khan AU, Mei YH, Wilson T (1992). A proposed function for spermine and spermidine: protection of replicating DNA against damage by singlet oxygen. Proc Natl Acad Sci U S A.

[12] Ha HC, Sirisoma NS, Kuppusamy P, Zweier JL, Woster PM, Casero RA (1998). The natural polyamine spermine functions directly as a free radical scavenger. Proc Natl Acad Sci U S A.

[13] Ha HC, Yager JD, Woster PA, Casero RA (1998). Structural specificity of polyamines and polyamine analogues in the protection of DNA from strand breaks induced by reactive oxygen species. Biochem Biophys Res Commun.

[14] Das KC, Misra HP (2004). Hydroxyl radical scavenging and singlet oxygen quenching properties of polyamines. Mol Cell Biochem.

[15] Fujisawa S, Kadoma Y (2005). Kinetic evaluation of polyamines as radical scavengers. Anticancer Res.

[16] Pegg AE (2013). Toxicity of polyamines and their metabolic products. Chem Res Toxicol.

[17] Bianchi M, Bellini A, Cervelli M, Degan P, Marcocci L, Martini F, Scatteia M, Mariottini P, Amendola R (2007). Chronic sub-lethal oxidative stress by spermine oxidase overactivity induces continuous DNA repair and hypersensitivity to radiation exposure. Biochim Biophys Acta.

[18] Amendola R, Cervelli M, Fratini E, Polticelli F, Sallustio DE, Mariottini P (2009). Spermine metabolism and anticancer therapy. Curr Cancer Drug Targets.

[19] Amendola R, Cervelli M, Fratini E, Sallustio DE, Tempera G, Ueshima T, Mariottini P, Agostinelli E (2013). Reactive oxygen species spermine metabolites generated from amine oxidases and radiation represent a therapeutic gain in cancer treatments. Int J Oncol.

[20] Perrone C, Pomella S, Cassandri M, Pezzella M, Giuliani S, Gasperi T, Porrazzo A, Alisi A, Pastore A, Codenotti S, Fanzani A, Barillari G, Conti LA, De Angelis B, Quintarelli C, Mariottini P, Locatelli F, Marampon F, Rota R, Cervelli M (2023). Spermine oxidase induces DNA damage and sensitizes fusion negative rhabdomyosarcoma cells to irradiation. Front Cell Dev Biol.

[21] Williams K (1997). Interactions of polyamines with ion channels. Biochem J.

[22] Wallace HM, Fraser AV, Hughes A (2003). A perspective of polyamine metabolism. Biochem J.

[23] Jänne J, Alhonen L, Pietilä M, Keinänen TA (2004). Genetic approaches to the cellular functions of polyamines in mammals. Eur J Biochem.

[24] Bowie D (2018). Polyamine-mediated channel block of ionotropic glutamate receptors and its regulation by auxiliary proteins. J Biol Chem.

[25] Stanfield PR, Sutcliffe MJ (2003). Spermine is fit to block inward rectifier (Kir) channels. J Gen Physiol.

[26] Dhara M, Matta JA, Lei M, Knowland D, Yu H, Gu S, Bredt DS (2020). Polyamine regulation of ion channel assembly and implications for nicotinic acetylcholine receptor pharmacology. Nat Commun.

[27] Makletsova MG, Rikhireva GT, Kirichenko EY, Trinitatsky IY, Vakulenko MY, Ermakov AM (2022). The role of polyamines in the mechanisms of cognitive impairment. Neurochem J.

[28] Shin WW, Fong WF, Pang SF, Wong PC (1985). Limited blood-brain barrier transport of polyamines. J Neurochem.

[29] Rieck J, Skatchkov SN, Derst C, Eaton MJ, Veh RW (2022). Unique chemistry, intake, and metabolism of polyamines in the central nervous system (CNS) and its body. Biomolecules.

[30] Cervelli M, Averna M, Vergani L, Pedrazzi M, Amato S, Fiorucci C, Rossi MN, Maura G, Mariottini P, Cervetto C, Marcoli M (2022). The involvement of polyamines catabolism in the crosstalk between neurons and astrocytes in neurodegeneration. Biomedicines.

[31] Kambis TN, Tofilau HMN, Gawargi FI, Chandra S, Mishra PK (2021). Regulating polyamine metabolism by miRNAs in diabetic cardiomyopathy. Curr Diab Rep.

[32] Tauc M, Cougnon M, Carcy R, Melis N, Hauet T, Pellerin L, Blondeau N, Pisani DF (2021). The eukaryotic initiation factor 5A (eIF5A1), the molecule, mechanisms and recent insights into the pathophysiological roles. Cell Biosci.

[33] Padgett LR, Robertson MA, Anderson-Baucum EK, Connors CT, Wu W, Mirmira RG, Mastracci TL (2021). Deoxyhypusine synthase, an essential enzyme for hypusine biosynthesis, is required for proper exocrine pancreas development. Faseb J.

[34] Kulkarni A, Anderson CM, Mirmira RG, Tersey SA (2022). Role of polyamines and hypusine in Β cells and diabetes pathogenesis. Metabolites.

[35] Ramos-Molina B, Queipo-Ortuño MI, Lambertos A, Tinahones FJ, Peñafiel R (2019). Dietary and gut microbiota polyamines in obesity- and age-related diseases. Front Nutr.

[36] Hayashi Y, Tanaka J, Morizumi Y, Kitamura Y, Hattori Y (2004). Polyamine levels in brain and plasma after acute restraint or water-immersion restraint stress in mice. Neurosci Lett.

[37] Ceci R, Duranti G, Giuliani S, Rossi MN, Dimauro I, Sabatini S, Mariottini P, Cervelli M (2022). The impact of spermidine on C2c12 myoblasts proliferation, redox status and polyamines metabolism under H_2_O_2_ exposure. Int J Mol Sci.

[38] Cervelli M, Angelucci E, Germani F, Amendola R, Mariottini P (2014). Inflammation, carcinogenesis and neurodegeneration studies in transgenic animal models for polyamine research. Amino Acids.

[39] Cervelli M, Leonetti A, Duranti G, Sabatini S, Ceci R, Mariottini P (2018). Skeletal muscle pathophysiology: the emerging role of spermine oxidase and spermidine. Med Sci (Basel).

[40] Nakanishi S, Cleveland JL (2021). Polyamine homeostasis in development and disease. Med Sci (Basel).

[41] Shantz LM, Levin VA (2007). Regulation of ornithine decarboxylase during oncogenic transformation: mechanisms and therapeutic potential. Amino Acids.

[42] Ramos-Molina B, Lambertos A, Peñafiel R (2018). Antizyme inhibitors in polyamine metabolism and beyond: physiopathological implications. Med Sci.

[43] Murakami Y, Matsufuji S, Kameji T, Hayashi S, Igarashi K, Tamura T, Tanaka K, Ichihara A (1992). Ornithine decarboxylase is degraded by the 26s proteasome without ubiquitination. Nature.

[44] Kahana C (2009). Antizyme and antizyme inhibitor, a regulatory tango. Cell Mol Life Sci.

[45] López-Contreras AJ, Ramos-Molina B, Cremades A, Peñafiel R (2010). Antizyme inhibitor 2: molecular, cellular and physiological aspects. Amino Acids.

[46] Polticelli F, Salvi D, Mariottini P, Amendola R, Cervelli M (2012). Molecular evolution of the polyamine oxidase gene family in Metazoa. BMC Evol Biol.

[47] Cervelli M, Salvi D, Polticelli F, Amendola R, Mariottini P (2013). Structure-function relationships in the evolutionary framework of spermine oxidase. J Mol Evol.

[48] Cervelli M, Polticelli F, Federico R, Mariottini P (2003). Heterologous expression and characterization of mouse spermine oxidase. J Biol Chem.

[49] Tavladoraki P, Cervelli M, Antonangeli F, Minervini G, Stano P, Federico R, Mariottini P, Polticelli F (2011). Probing mammalian spermine oxidase enzyme-substrate complex through molecular modeling, site-directed mutagenesis and biochemical characterization. Amino Acids.

[50] Uemura T, Gerner EW (2011). Polyamine transport systems in mammalian cells and tissues. Methods Mol Biol.

[51] Belting M, Mani K, Jönsson M, Cheng F, Sandgren S, Jonsson S, Ding K, Delcros JG, Fransson LA (2003). Glypican-1 Is a vehicle for polyamine uptake in mammalian cells: a pivotal role for nitrosothiol-derived nitric oxide. J Biol Chem.

[52] Uemura T, Stringer DE, Blohm-Mangone KA, Gerner EW (2010). Polyamine transport is mediated by both endocytic and solute carrier transport mechanisms in the gastrointestinal tract. Am J Physiol Gastrointest Liver Physiol.

[53] Azfar M, van Veen S, Houdou M, Hamouda NN, Eggermont J, Vangheluwe P (2022). P5b-ATPases in the mammalian polyamine transport system and their role in disease. Biochim Biophys Acta Mol Cell Res.

[54] Hiasa M, Miyaji T, Haruna Y, Takeuchi T, Harada Y, Moriyama S, Yamamoto A, Omote H, Moriyama Y (2014). Identification of a mammalian vesicular polyamine transporter. Sci Rep.

[55] Uemura T, Yerushalmi HF, Tsaprailis G, Stringer DE, Pastorian KE, Hawel L, Byus CV, Gerner EW (2008). Identification and characterization of a diamine exporter in colon epithelial cells. J Biol Chem.

[56] Daigle ND, Carpentier GA, Frenette-Cotton R, Simard MG, Lefoll M-H, Noël M, Caron L, Noël J, Isenring P (2009). Molecular characterization of a human cation-Cl−cotransporter (SLC12A8A, CCC9A) that promotes polyamine and amino acid transport. J Cell Physiol.

[57] Hamouda NN, Van den Haute C, Vanhoutte R, Sannerud R, Azfar M, Mayer R, Cortés Calabuig Á, Swinnen JV, Agostinis P, Baekelandt V, Annaert W, Impens F, Verhelst SHL, Eggermont J, Martin S, Vangheluwe P (2021). ATP13A3 is a major component of the enigmatic mammalian polyamine transport system. J Biol Chem.

[58] Li P, Wang K, Salustros N, Grønberg C, Gourdon P (2021). Structure and transport mechanism of P5b-ATPases. Nat Commun.

[59] Mitchell JL, Judd GG, Bareyal-Leyser A, Ling SY (1994). Feedback repression of polyamine transport is mediated by antizyme in mammalian tissue-culture cells. Biochem J.

[60] López-Contreras AJ, Ramos-Molina B, Cremades A, Peñafiel R (2008). Antizyme inhibitor 2 (AZIN2/ODCp) stimulates polyamine uptake in mammalian cells. J Biol Chem.

[61] Igarashi K, Kashiwagi K (2000). Polyamines: mysterious modulators of cellular functions. Biochem Biophys Res Commun.

[62] Casero RA, Pegg AE (2009). Polyamine catabolism and disease. Biochem J.

[63] Madeo F, Eisenberg T, Pietrocola F, Kroemer G (2018). Spermidine in health and disease. Science.

[64] Fernandez-Garcia JC, Delpino-Rius A, Samarra I, Castellano-Castillo D, Muñoz-Garach A, Bernal-Lopez MR, Queipo-Ortuño MI, Cardona F, Ramos-Molina B, Tinahones FJ (2019). Type 2 diabetes is associated with a different pattern of serum polyamines: a case-control study from the PREDIMED-plus trial. J Clin Med.

[65] Sims EK, Kulkarni A, Hull A, Woerner SE, Cabrera S, Mastrandrea LD, Hammoud B, Sarkar S, Nakayasu ES, Mastracci TL, Perkins SM, Ouyang F, Webb-Robertson BJ, Enriquez JR, Tersey SA, Evans-Molina C, Long SA, Blanchfield L, Gerner EW, Mirmira RG, DiMeglio LA (2023). Inhibition of polyamine biosynthesis preserves Β cell function in type 1 diabetes. Cell Rep Med.

[66] Zhang Y, Zhang TT, Gao L, Tan YN, Li YT, Tan XY, Huang TX, Li HH, Bai F, Zou C, Pei XH, Tan BB, Fu L (2022). Downregulation of MTAP promotes tumor growth and metastasis by regulating ODC activity in breast cancer. Int J Biol Sci.

[67] Pérez-Riesgo E, Hernando-Pérez E, Feijóo V, Tajada S, Núñez L, Villalobos C (2023). Transcriptional basis of Ca^2+^ remodeling reversal induced by polyamine synthesis inhibition in colorectal cancer cells. Cancers (Basel).

[68] Ou Y, Wang SJ, Li D, Chu B, Gu W (2016). Activation of SAT1 engages polyamine metabolism with P53-mediated ferroptotic responses. Proc Natl Acad Sci U S A.

[69] Thakur VS, Aguila B, Brett-Morris A, Creighton CJ, Welford SM (2019). Spermidine/spermine N1-acetyltransferase 1 is a gene-specific transcriptional regulator that drives brain tumor aggressiveness. Oncogene.

[70] Xuan M, Gu X, Li J, Huang D, Xue C, He Y (2023). Polyamines: their significance for maintaining health and contributing to diseases. Cell Commun Signal.

[71] Olsen RR, Zetter BR (2011). Evidence of a role for antizyme and antizyme inhibitor as regulators of human cancer. Mol Cancer Res.

[72] Chen L, Li Y, Lin CH, Chan TH, Chow RK, Song Y, Liu M, Yuan YF, Fu L, Kong KL, Qi L, Li Y, Zhang N, Tong AH, Kwong DL, Man K, Lo CM, Lok S, Tenen DG, Guan XY (2013). Recoding RNA editing of AZIN1 predisposes to hepatocellular carcinoma. Nat Med.

[73] Qiu S, Liu J, Xing F (2017). Antizyme inhibitor 1: a potential carcinogenic molecule. Cancer Sci.

[74] Cason AL, Ikeguchi Y, Skinner C, Wood TC, Holden KR, Lubs HA, Martinez F, Simensen RJ, Stevenson RE, Pegg AE, Schwartz CE (2003). X-linked spermine synthase gene (SMS) defect: the first polyamine deficiency syndrome. Eur J Hum Genet.

[75] Bupp C, Michael J, VanSickle E, Rajasekaran S, Bachmann AS (2022) Bachmann-Bupp Syndrome. GeneReviews® [Internet]

[76] Ramirez A, Heimbach A, Gründemann J, Stiller B, Hampshire D, Cid LP, Goebel I, Mubaidin AF, Wriekat AL, Roeper J, Al-Din A, Hillmer AM, Karsak M, Liss B, Woods CG, Behrens MI, Kubisch C (2006). Hereditary parkinsonism with dementia is caused by mutations in ATP13A2, encoding a lysosomal type 5 P-type ATPase. Nat Genet.

[77] Lewandowski NM, Ju S, Verbitsky M, Ross B, Geddie ML, Rockenstein E, Adame A, Muhammad A, Vonsattel JP, Ringe D, Cote L, Lindquist S, Masliah E, Petsko GA, Marder K, Clark LN, Small SA (2010). Polyamine pathway contributes to the pathogenesis of Parkinson disease. Proc Natl Acad Sci U S A.

[78] van Veen S, Martin S, Van den Haute C, Benoy V, Lyons J, Vanhoutte R, Kahler JP, Decuypere JP, Gelders G, Lambie E, Zielich J, Swinnen JV, Annaert W, Agostinis P, Ghesquière B, Verhelst S, Baekelandt V, Eggermont J, Vangheluwe P (2020). ATP13A2 deficiency disrupts lysosomal polyamine export. Nature.

[79] Mäkitie LT, Kanerva K, Polvikoski T, Paetau A, Andersson LC (2010). Brain neurons express ornithine decarboxylase-activating antizyme inhibitor 2 with accumulation in Alzheimer’s disease. Brain Pathol.

[80] Sandusky-Beltran LA, Kovalenko A, Placides DS, Ratnasamy K, Ma C, Hunt JB, Liang H, Calahatian JIT, Michalski C, Fahnestock M, Blair LJ, Darling AL, Baker JD, Fontaine SN, Dickey CA, Gamsby JJ, Nash KR, Abner E, Selenica MB, Lee DC (2021). Aberrant AZIN2 and polyamine metabolism precipitates tau neuropathology. J Clin Invest.

[81] Lambertos A, Nuñez-Sanchez MA, López-García C, López-Contreras AJ, Ramos-Molina B, Peñafiel R (2023). Antizyme inhibitor 2-deficient mice exhibit altered brain polyamine levels and reduced locomotor activity. Biomolecules.

[82] Inouye M, Delihas N (1988). Small RNAs in the prokaryotes: a growing list of diverse roles. Cell.

[83] Carninci P, Kasukawa T, Katayama S, Gough J, Frith MC, Maeda N, Oyama R, Ravasi T, Lenhard B, Wells C, Kodzius R, Shimokawa K, Bajic VB, Brenner SE, Batalov S, Forrest AR, Zavolan M, Davis MJ, Wilming LG, Aidinis V, Allen JE, Ambesi-Impiombato A, Apweiler R, Aturaliya RN, Bailey TL, Bansal M, Baxter L, Beisel KW, Bersano T, Bono H, Chalk AM, Chiu KP, Choudhary V, Christoffels A, Clutterbuck DR, Crowe ML, Dalla E, Dalrymple BP, de Bono B, Della Gatta G, di Bernardo D, Down T, Engstrom P, Fagiolini M, Faulkner G, Fletcher CF, Fukushima T, Furuno M, Futaki S, Gariboldi M, Georgii-Hemming P, Gingeras TR, Gojobori T, Green RE, Gustincich S, Harbers M, Hayashi Y, Hensch TK, Hirokawa N, Hill D, Huminiecki L, Iacono M, Ikeo K, Iwama A, Ishikawa T, Jakt M, Kanapin A, Katoh M, Kawasawa Y, Kelso J, Kitamura H, Kitano H, Kollias G, Krishnan SP, Kruger A, Kummerfeld SK, Kurochkin IV, Lareau LF, Lazarevic D, Lipovich L, Liu J, Liuni S, McWilliam S, Madan Babu M, Madera M, Marchionni L, Matsuda H, Matsuzawa S, Miki H, Mignone F, Miyake S, Morris K, Mottagui-Tabar S, Mulder N, Nakano N, Nakauchi H, Ng P, Nilsson R, Nishiguchi S, Nishikawa S, Nori F, Ohara O, Okazaki Y, Orlando V, Pang KC, Pavan WJ, Pavesi G, Pesole G, Petrovsky N, Piazza S, Reed J, Reid JF, Ring BZ, Ringwald M, Rost B, Ruan Y, Salzberg SL, Sandelin A, Schneider C, Schönbach C, Sekiguchi K, Semple CA, Seno S, Sessa L, Sheng Y, Shibata Y, Shimada H, Shimada K, Silva D, Sinclair B, Sperling S, Stupka E, Sugiura K, Sultana R, Takenaka Y, Taki K, Tammoja K, Tan SL, Tang S, Taylor MS, Tegner J, Teichmann SA, Ueda HR, van Nimwegen E, Verardo R, Wei CL, Yagi K, Yamanishi H, Zabarovsky E, Zhu S, Zimmer A, Hide W, Bult C, Grimmond SM, Teasdale RD, Liu ET, Brusic V, Quackenbush J, Wahlestedt C, Mattick JS, Hume DA, Kai C, Sasaki D, Tomaru Y, Fukuda S, Kanamori-Katayama M, Suzuki M, Aoki J, Arakawa T, Iida J, Imamura K, Itoh M, Kato T, Kawaji H, Kawagashira N, Kawashima T, Kojima M, Kondo S, Konno H, Nakano K, Ninomiya N, Nishio T, Okada M, Plessy C, Shibata K, Shiraki T, Suzuki S, Tagami M, Waki K, Watahiki A, Okamura-Oho Y, Suzuki H, Kawai J, Hayashizaki Y (2005). The transcriptional landscape of the mammalian genome. Science.

[84] Amaral PP, Dinger ME, Mercer TR, Mattick JS (2008). The eukaryotic genome as an RNA machine. Science.

[85] Zhou X, Wu X, Lai K, Zhou R, Chen Z, Yang Z, Gao X (2023). Discovery of the hidden coding information in cancers: mechanisms and biological functions. Int J Cancer.

[86] Taft RJ, Pheasant M, Mattick JS (2007). The relationship between non-protein-coding DNA and eukaryotic complexity. BioEssays.

[87] Heimberg AM, Sempere LF, Moy VN, Donoghue PC, Peterson KJ (2008). MicroRNAs and the advent of vertebrate morphological complexity. Proc Natl Acad Sci U S A.

[88] Ponting CP, Oliver PL, Reik W (2009). Evolution and functions of long noncoding RNAs. Cell.

[89] Rossi MN, Antonangeli F (2014). LncRNAs: new players in apoptosis control. Int J Cell Biol.

[90] Ahadi A (2021). A systematic review of microRNAs as potential biomarkers for diagnosis and prognosis of gastric cancer. Immunogenetics.

[91] Rinn JL, Kertesz M, Wang JK, Squazzo SL, Xu X, Brugmann SA, Goodnough LH, Helms JA, Farnham PJ, Segal E, Chang HY (2007). Functional demarcation of active and silent chromatin domains in human HOX loci by noncoding RNAs. Cell.

[92] Rybak-Wolf A, Stottmeister C, Glažar P, Jens M, Pino N, Giusti S, Hanan M, Behm M, Bartok O, Ashwal-Fluss R, Herzog M, Schreyer L, Papavasileiou P, Ivanov A, Öhman M, Refojo D, Kadener S, Rajewsky N (2015). Circular RNAs in the mammalian brain are highly abundant, conserved, and dynamically expressed. Mol Cell.

[93] Andresini O, Rossi MN, Matteini F, Petrai S, Santini T, Maione R (2019). The long non-coding RNA Kcnq1ot1 controls maternal p57 expression in muscle cells by promoting H3K27me3 accumulation to an intragenic MyoD-binding region. Epigenet Chromatin.

[94] Kiltschewskij D, Cairns MJ (2019). Temporospatial guidance of activity-dependent gene expression by microRNA: mechanisms and functional implications for neural plasticity. Nucleic Acids Res.

[95] Starega-Roslan J, Koscianska E, Kozlowski P, Krzyzosiak WJ (2011). The role of the precursor structure in the biogenesis of microRNA. Cell Mol Life Sci.

[96] Macfarlane LA, Murphy PR (2010). MicroRNA: biogenesis, function and role in cancer. Curr Genomics.

[97] Okamura K, Ishizuka A, Siomi H, Siomi MC (2004). Distinct roles for Argonaute proteins in small RNA-directed RNA cleavage pathways. Genes Dev.

[98] Kosik KS (2006). The neuronal microRNA system. Nat Rev Neurosci.

[99] Chalfie M, Horvitz HR, Sulston JE (1981). Mutations that lead to reiterations in the cell lineages of C. Elegans Cell.

[100] Ambros V (2004). The functions of animal microRNAs. Nature.

[101] Gebert LFR, MacRae IJ (2019). Regulation of microRNA function in animals. Nat Rev Mol Cell Biol.

[102] Rinn JL, Chang HY (2012). Genome regulation by long noncoding RNAs. Annu Rev Biochem.

[103] Wang X, Arai S, Song X, Reichart D, Du K, Pascual G, Tempst P, Rosenfeld MG, Glass CK, Kurokawa R (2008). Induced ncRNAs allosterically modify RNA-binding proteins in cis to inhibit transcription. Nature.

[104] Huarte M, Rinn JL (2010). Large non-coding RNAs: missing links in cancer?. Hum Mol Genet.

[105] Kino T, Hurt DE, Ichijo T, Nader N, Chrousos GP (2010). Noncoding RNA Gas5 is a growth arrest- and starvation-associated repressor of the glucocorticoid receptor. Sci Signal.

[106] Cesana M, Cacchiarelli D, Legnini I, Santini T, Sthandier O, Chinappi M, Tramontano A, Bozzoni I (2011). A long noncoding RNA controls muscle differentiation by functioning as a competing endogenous RNA. Cell.

[107] Fatica A, Bozzoni I (2014). Long non-coding RNAs: new players in cell differentiation and development. Nat Rev Genet.

[108] Lasda E, Parker R (2014). Circular RNAs: diversity of form and function. RNA.

[109] Mehta SL, Chokkalla AK, Vemuganti R (2021). Noncoding RNA crosstalk in brain health and diseases. Neurochem Int.

[110] Memczak S, Jens M, Elefsinioti A, Torti F, Krueger J, Rybak A, Maier L, Mackowiak SD, Gregersen LH, Munschauer M, Loewer A, Ziebold U, Landthaler M, Kocks C, le Noble F, Rajewsky N (2013). Circular RNAs are a large class of animal RNAs with regulatory potency. Nature.

[111] Chen L-L (2020). The expanding regulatory mechanisms and cellular functions of circular RNAs. Nat Rev Mol Cell Biol.

[112] You X, Vlatkovic I, Babic A, Will T, Epstein I, Tushev G, Akbalik G, Wang M, Glock C, Quedenau C, Wang X, Hou J, Liu H, Sun W, Sambandan S, Chen T, Schuman EM, Chen W (2015). Neural circular RNAs are derived from synaptic genes and regulated by development and plasticity. Nat Neurosci.

[113] Mehta SL, Dempsey RJ, Vemuganti R (2020). Role of circular RNAs in brain development and CNS diseases. Prog Neurobiol.

[114] Cheng J, Li G, Wang W, Stovall DB, Sui G, Li D (2023). Circular RNAs with protein-coding ability in oncogenesis. Biochim Biophys Acta Rev Cancer.

[115] Novita Sari I, Setiawan T, Seock Kim K, Toni Wijaya Y, Won Cho K, Young Kwon H (2021). Metabolism and function of polyamines in cancer progression. Cancer Lett.

[116] Fahrmann JF, Vykoukal J, Fleury A, Tripathi S, Dennison JB, Murage E, Wang P, Yu CY, Capello M, Creighton CJ, Do KA, Long JP, Irajizad E, Peterson C, Katayama H, Disis ML, Arun B, Hanash S (2020). Association between plasma diacetylspermine and tumor spermine synthase with outcome in triple-negative breast cancer. J Natl Cancer Inst.

[117] Chen C, Pan Y, Bai L, Chen H, Duan Z, Si Q, Zhu R, Chuang T-H, Luo Y (2021). MicroRNA-3613-3p functions as a tumor suppressor and represents a novel therapeutic target in breast cancer. Breast Cancer Res.

[118] Liu N, Zhang T, Steer CJ, Song G (2022). MicroRNA-378a-3p prevents initiation and growth of colorectal cancer by fine tuning polyamine synthesis. Cell Biosci.

[119] Li Y, Gong P, Hou J-x, Huang W, Ma X-p, Wang Y-l, Li J, Cui X-b, Li N (2018). miR-34a regulates multidrug resistance via positively modulating OAZ2 signaling in colon cancer cells. J Immunol Res.

[120] Murray-Stewart T, Sierra JC, Piazuelo MB, Mera RM, Chaturvedi R, Bravo LE, Correa P, Schneider BG, Wilson KT, Casero RA (2016). Epigenetic silencing of miR-124 prevents spermine oxidase regulation: implications for helicobacter pylori-induced gastric cancer. Oncogene.

[121] Jichao W, Jing G, Fei W, Lei C, Qian L, Jie F, Hongyun W, Hua G, Yazhuo Z (2019). miRNA-199a-5p functions as a tumor suppressor in prolactinomas. Open Chem.

[122] Nakada C, Matsuura K, Tsukamoto Y, Tanigawa M, Yoshimoto T, Narimatsu T, Nguyen LT, Hijiya N, Uchida T, Sato F, Mimata H, Seto M, Moriyama M (2008). Genome-wide microRNA expression profiling in renal cell carcinoma: significant down-regulation of miR-141 and miR-200c. J Pathol.

[123] Nakada C, Hijiya N, Tsukamoto Y, Yano S, Kai T, Uchida T, Kimoto M, Takahashi M, Daa T, Matsuura K, Shin T, Mimata H, Moriyama M (2020). A transgenic mouse expressing miR-210 in proximal tubule cells shows mitochondrial alteration: possible association of miR-210 with a shift in energy metabolism. J Pathol.

[124] Xiao Y, Li C, Wang H, Liu Y (2020). LINC00265 targets miR-382-5p to regulate SAT1, VAV3 and angiogenesis in osteosarcoma. Aging (Albany NY).

[125] Sui X, Hu N, Zhang Z, Wang Y, Wang P, Xiu G (2021). ASMTL-AS1 impedes the malignant progression of lung adenocarcinoma by regulating SAT1 to promote ferroptosis. Pathol Int.

[126] Meng X, Peng J, Xie X, Yu F, Wang W, Pan Q, Jin H, Huang X, Yu H, Li S, Feng D, Liu Q, Fang L, Lee MH (2022). Roles of lncRNA LVBU in regulating urea cycle/polyamine synthesis axis to promote colorectal carcinoma progression. Oncogene.

[127] Yang W-S, Yeh WW, Campbell M, Chang L, Chang P-C (2021). Long non-coding RNA KIKAT/LINC01061 as a novel epigenetic regulator that relocates KDM4A on chromatin and modulates viral reactivation. PLoS Pathog.

[128] Genedani S, Saltini S, Benelli A, Filaferro M, Bertolini A (2001). Influence of same on the modifications of brain polyamine levels in an animal model of depression. NeuroReport.

[129] Fiori LM, Bureau A, Labbe A, Croteau J, Noël S, Mérette C, Turecki G (2011). Global gene expression profiling of the polyamine system in suicide completers. Int J Neuropsychopharmacol.

[130] Cervetto C, Vergani L, Passalacqua M, Ragazzoni M, Venturini A, Cecconi F, Berretta N, Mercuri N, D’Amelio M, Maura G, Mariottini P, Voci A, Marcoli M, Cervelli M (2016). Astrocyte-dependent vulnerability to excitotoxicity in spermine oxidase-overexpressing mouse. NeuroMol Med.

[131] Leonetti A, Baroli G, Fratini E, Pietropaoli S, Marcoli M, Mariottini P, Cervelli M (2020). Epileptic seizures and oxidative stress in a mouse model over-expressing spermine oxidase. Amino Acids.

[132] Cervetto C, Averna M, Vergani L, Pedrazzi M, Amato S, Pelassa S, Giuliani S, Baldini F, Maura G, Mariottini P, Marcoli M, Cervelli M (2021). Reactive astrocytosis in a mouse model of chronic polyamine catabolism activation. Biomolecules.

[133] Liu J, Yu Z, Maimaiti B, Meng Q, Meng H (2022). The potential role of polyamines in epilepsy and epilepsy-related pathophysiological changes. Biomolecules.

[134] Zhang D, Zhao T, Ang HS, Chong P, Saiki R, Igarashi K, Yang H, Vardy LA (2012). AMD1 is essential for ESC self-renewal and is translationally down-regulated on differentiation to neural precursor cells. Genes Dev.

[135] Gross JA, Turecki G (2013). Suicide and the polyamine system. CNS Neurol Disord Drug Targets.

[136] Pietropaoli S, Leonetti A, Cervetto C, Venturini A, Mastrantonio R, Baroli G, Persichini T, Colasanti M, Maura G, Marcoli M, Mariottini P, Cervelli M (2018). Glutamate excitotoxicity linked to spermine oxidase overexpression. Mol Neurobiol.

[137] Baroli G, Sanchez JR, Agostinelli E, Mariottini P, Cervelli M (2020). Polyamines: the possible missing link between mental disorders and epilepsy (review). Int J Mol Med.

[138] Lopez JP, Fiori LM, Gross JA, Labonte B, Yerko V, Mechawar N, Turecki G (2014). Regulatory role of miRNAs in polyamine gene expression in the prefrontal cortex of depressed suicide completers. Int J Neuropsychopharmacol.

[139] Wang G, Han B, Shen L, Wu S, Yang L, Liao J, Wu F, Li M, Leng S, Zang F, Zhang Y, Bai Y, Mao Y, Chen B, Yao H (2020). Silencing of circular RNA HIPK2 in neural stem cells enhances functional recovery following ischaemic stroke. EBioMedicine.

[140] Fan J, Chen M, Wang X, Tian Z, Wang J, Fan D, Zeng J, Zhang K, Dai X (2019). Targeting Smox is neuroprotective and ameliorates brain inflammation in cerebral ischemia/reperfusion rats. Toxicol Sci.

[141] Wang Y, Chen J, Li S, Zhang X, Guo Z, Hu J, Shao X, Song N, Zhao Y, Li H, Yang G, Xu C, Wei C (2020). Exogenous spermine attenuates rat diabetic cardiomyopathy via suppressing ROS-p53 mediated downregulation of calcium-sensitive receptor. Redox Biol.

[142] Bertero T, Handen AL, Chan SY (2018). Factors associated with heritable pulmonary arterial hypertension exert convergent actions on the miR-130/301-vascular matrix feedback loop. Int J Mol Sci.

[143] Wynn TA, Ramalingam TR (2012). Mechanisms of fibrosis: therapeutic translation for fibrotic disease. Nat Med.

[144] Paris AJ, Snapir Z, Christopherson CD, Kwok SY, Lee UE, Ghiassi-Nejad Z, Kocabayoglu P, Sninsky JJ, Llovet JM, Kahana C, Friedman SL (2011). A polymorphism that delays fibrosis in hepatitis C promotes alternative splicing of AZIN1. Reducing Fibrogen Hepatol.

[145] Tao L, Bei Y, Chen P, Lei Z, Fu S, Zhang H, Xu J, Che L, Chen X, Sluijter JP, Das S, Cretoiu D, Xu B, Zhong J, Xiao J, Li X (2016). Crucial role of miR-433 in regulating cardiac fibrosis. Theranostics.

[146] Li R, Chung ACK, Dong Y, Yang W, Zhong X, Lan HY (2013). The microRNA miR-433 promotes renal fibrosis by amplifying the TGF-β/Smad3-Azin1 pathway. Kidney Int.

[147] Weber KT, Sun Y, Bhattacharya SK, Ahokas RA, Gerling IC (2013). Myofibroblast-mediated mechanisms of pathological remodelling of the heart. Nat Rev Cardiol.

[148] Gyöngyösi M, Winkler J, Ramos I, Do QT, Firat H, McDonald K, González A, Thum T, Díez J, Jaisser F, Pizard A, Zannad F (2017). Myocardial fibrosis: biomedical research from bench to bedside. Eur J Heart Fail.

[149] Zhu Y, Pan W, Yang T, Meng X, Jiang Z, Tao L, Wang L (2019). Upregulation of circular RNA circNFIB attenuates cardiac fibrosis by sponging miR-433. Front Genet.

[150] Concepcion CP, Bonetti C, Ventura A (2012). The microRNA-17-92 family of microRNA clusters in development and disease. Cancer J.

[151] Konishi T, Lentsch AB (2017). Hepatic ischemia/reperfusion: mechanisms of tissue injury, repair, and regeneration. Gene Expr.

[152] Sun Q, Gong J, Gong X, Wu J, Hu Z, Zhang Q, Zhu X (2022). Long non-coding RNA MALAT1 aggravated liver ischemia-reperfusion injury via targeting miR-150-5p/AZIN1. Bioengineered.

[153] Liang T, Xie J, Zhao J, Huang W, Xu Z, Tian P, Mi C, Dai M, Zhang S, Zhang H (2021). Novel lnc-HZ03 and miR-HZ03 promote BPDE-induced human trophoblastic cell apoptosis and induce miscarriage by upregulating p53/SAT1 pathway. Cell Biol Toxicol.

[154] Mehta SL, Pandi G, Vemuganti R (2017). Circular RNA expression profiles alter significantly in mouse brain after transient focal ischemia. Stroke.

[155] Weng J, Zhang P, Yin X, Jiang B (2018). The whole transcriptome involved in denervated muscle atrophy following peripheral nerve injury. Front Mol Neurosci.

[156] Reinoso-Sánchez JF, Baroli G, Duranti G, Scaricamazza S, Sabatini S, Valle C, Morlando M, Casero RA, Bozzoni I, Mariottini P, Ceci R, Cervelli M (2020). Emerging role for linear and circular spermine oxidase RNAs in skeletal muscle physiopathology. Int J Mol Sci.

[157] Han Z, Mou Z, Jing Y, Jiang R, Sun T (2023). CircSmox knockdown alleviates PC12 cell apoptosis and inflammation in spinal cord injury by miR-340-5p/Smurf1 axis. Immun Inflamm Dis.

[158] Shao L, Liu X, Zhu S, Liu C, Gao Y, Xu X (2018). The role of Smurf1 in neuronal necroptosis after lipopolysaccharide-induced neuroinflammation. Cell Mol Neurobiol.

[159] Yan J, Ai C, Chen Q, Wang Q, Zhu Y, Li M, Chen K, He M, Shen M, Chen L, Zhang R, Zheng C, Liao W, Bin J, Lin H, Ma S, Tan N, Liao Y (2023) CircMap4k2 reactivated by aneurysm plication alleviates residual cardiac remodeling after SVR by enhancing cardiomyocyte proliferation in post-MI mice. J Adv Res10.1016/j.jare.2023.11.03438043608

[160] Jeck WR, Sorrentino JA, Wang K, Slevin MK, Burd CE, Liu J, Marzluff WF, Sharpless NE (2013). Circular RNAs are abundant, conserved, and associated with ALU repeats. RNA.

[161] Salzman J, Chen RE, Olsen MN, Wang PL, Brown PO (2013). Cell-type specific features of circular RNA expression. PLoS Genet.

[162] Maass PG, Glažar P, Memczak S, Dittmar G, Hollfinger I, Schreyer L, Sauer AV, Toka O, Aiuti A, Luft FC, Rajewsky N (2017). A map of human circular RNAs in clinically relevant tissues. J Mol Med (Berl).

